# GLP-1 receptor agonists as promising disease-modifying agents in WFS1 spectrum disorder

**DOI:** 10.3389/fcdhc.2023.1171091

**Published:** 2023-06-02

**Authors:** Eleonora Panfili, Giulio Frontino, Maria Teresa Pallotta

**Affiliations:** ^1^ Department of Medicine and Surgery, University of Perugia, Perugia, Italy; ^2^ Diabetes Research Institute, Istituto di Ricovero e Cura a Carattere Scientifico (IRCCS) San Raffaele Hospital, Milano, Italy

**Keywords:** Wolfram syndrome type 1, wolframin (WFS1), GLP-1RAs, diabetes, neurodegeneration

## Abstract

WFS1 spectrum disorder (WFS1-SD) is a rare monogenic neurodegenerative disorder whose cardinal symptoms are childhood-onset diabetes mellitus, optic atrophy, deafness, diabetes insipidus, and neurological signs ranging from mild to severe. The prognosis is poor as most patients die prematurely with severe neurological disabilities such as bulbar dysfunction and organic brain syndrome. Mutation of the *WFS1* gene is recognized as the prime mover of the disease and responsible for a dysregulated ER stress signaling, which leads to neuron and pancreatic β-cell death. There is no currently cure and no treatment that definitively arrests the progression of the disease. GLP-1 receptor agonists appear to be an efficient way to reduce elevated ER stress *in vitro* and *in vivo*, and increasing findings suggest they could be effective in delaying the progression of WFS1-SD. Here, we summarize the characteristics of GLP-1 receptor agonists and preclinical and clinical data obtained by testing them in WFS1-SD as a feasible strategy for managing this disease.

## Introduction

Wolfram syndrome type 1 spectrum disorder (WFS1-SD, OMIM: 222300, 614296) is a rare multi-systemic monogenic disease that comprises classic WFS1 spectrum disorder and nonclassic WFS1 spectrum disorder. Classic WFS1-SD is an autosomal recessive progressive neurodegenerative disorder characterized by the onset of diabetes mellitus and optic atrophy before the age of 16 years. Additional manifestations may include variable hearing impairment/deafness, diabetes insipidus, neurologic abnormalities, neurogenic bladder, and psychiatric abnormalities. Nonclassic WFS1-SD is less common than classic WFS1-SD and is autosomal dominant. Phenotypes that appear to be milder than classic WFS1-SD include optic atrophy and hearing impairment; neonatal diabetes, profound congenital deafness, and cataracts; isolated diabetes mellitus; isolated congenital cataracts; and isolated congenital, slowly progressive, and low-frequency (<2000 Hz) sensorineural hearing loss (1). In both forms, the prognosis is poor with a median survival between 30 and 40 years. Death occurs usually as a consequence of severe neurological disabilities, mainly from respiratory failure caused by brain stem atrophy (2).

The causative gene of WFS1-SD is *WFS1*, contrarily from the less common Wolfram syndrome type 2 (WS2) which is caused by autosomal recessive mutations of the *CISD2* (CDGSH iron-sulfur domain-containing protein 2) gene. The two diseases also differ in symptoms, being WS2 characterized by bleeding, upper intestinal ulcer, defective platelet aggregation, and absence of diabetes insipidus and psychiatric disorders (3, 4).

*WFS1* codes for wolframin (WFS1), a transmembrane protein highly expressed in brain tissue, pancreatic β-cells, heart, lung, and placenta (5), and localized in the membrane of the endoplasmic reticulum (ER), where it plays a crucial role in maintaining ER homeostasis (4, 6, 7). ER is a cellular organelle responsible for the storage of Ca^2+^ ions, and for the correct folding and post-translational modification of several proteins (secretory proteins, cell surface receptors, integral membrane proteins, neurotransmitters, and hormones) (8). WFS1 loss of function causes an increase in the cytosolic concentration of Ca^2+^ ions, resulting in the establishment of chronic ER stress and the inappropriate activation of the unfolded protein response (UPR) signaling pathway (9). These events drive the cell to irreversible damage, which leads to apoptosis mainly in neuronal and pancreatic β-cells, where WFS1 expression is higher (7). Furthermore, it has been shown that WFS1 has a role in the transport of Ca^2+^ from the ER to the mitochondria and can therefore affect mitochondrial function (10).

Over 200 distinct mutations have been identified so far in WFS1-SD patients and new variants continue to be reported over time (11–13). Several attempts have been made to identify a genotype-phenotype correlation. However, the main difficulty in this regard relies on the existence of such a large number of variants of the WFS1 gene and the small size of patient cohorts because of the rarity of the disease (14–17).

Presently, no cure is available and until now patients with WFS1-SD have only profited from substitutive therapies for diabetes mellitus or diabetes insipidus (18). However, the identification of pathological molecular mechanisms has inspired new approaches, which mainly aim to restore Ca^2+^ homeostasis and contain ER stress to slow down the progression of the disease (4). Among the drugs tested *in vitro* and preclinical models, Valproate (NCT03717909) and Dantrolene (NCT02829268) were selected to undergo clinical trial investigation as possible therapeutic options for WFS1-SD (18). Increasing evidence from preclinical tests and off-label use suggests Glucagon-like peptide 1 receptor agonists (GLP-1RAs) as another possible therapeutic option.

### Glucagon-like peptide 1 and GLP-1 receptor agonists

Glucagon-like peptide 1 (GLP-1) is a hormone produced from L-cells of the small intestine by alternative processing of the proglucagon in response to nutrient ingestion (19, 20).

GLP-1 exerts its action through the GLP-1 receptor (GLP-1R), a member of G protein-coupled receptors (GPCRs). In particular, GLP-1 binds and activates a class B GPCRs coupled to Gs protein, leading to the activation of the enzyme adenyl cyclase and the consequent production of cyclic adenosine monophosphate (cAMP). Subsequently, several signal transduction pathways are initiated, generally involving protein kinase A (PKA) and exchange protein directly activated by cAMP (EPAC). Noteworthy, among the GLP-1 receptor-mediated signaling events, there is an increase in intracellular Ca^2+^ levels (19, 21).

GLP-1 is an incretin hormone and its main action is to improve glycemic control by stimulating glucose-dependent insulin secretion and promoting insulin synthesis (19, 20, 22). Furthermore, it inhibits glucagon release from α-cells (23) and preserves β-cell mass through the stimulation of their proliferation and inhibition of apoptosis (24–27). Moreover, GLP-1 has additional metabolic effects, namely delaying gastric emptying thereby inducing satiety, inducing central appetite suppression, and increasing natriuresis and diuresis (28–30). GLP-1 signaling in the brain transmits metabolic information to the neurons responsible for feeding behavior (31) but is also implicated in cognitive functions, such as learning and memory (32). It was likewise described a cardioprotective role for GLP-1, being capable to decrease blood pressure, improving microvascular function, and reducing inflammation (33). Notably, GLP-1 has a well-established central role since GLP-1Rs are expressed in the postrema area, hippocampus, accumbent nucleus, solitary tract nucleus, thalamus, afferent vagal system, and some other regions of the brain (34). GLP‐1 can be released from the intestine and pass through blood–brain barrier and affect the brain, but also GLP-1-producing neurons are present in several areas of the brain stem and hypothalamus (35). It is currently of great interest that GLP-1 has neuroprotective effects and can decrease inflammation and apoptosis (36–39). Several *in vivo* and *in vitro* studies using preclinical models of neurodegenerative diseases show that GLP-1R activation reduces the production of pro-inflammatory cytokines and immune cell infiltration in tissues (38, 40–42). Furthermore, it was determined that to induce the neuroprotective effects, the GLP-1R must be activated in the brain (38). Considering the numerous beneficial effects of GLP-1, this pleiotropic hormone is an attractive candidate for the treatment of obesity, diabetes, and neurodegenerative disorders. However, GLP-1 cannot be used in its native form because it has a very short half-life (1-2 min), being rapidly degraded by the enzyme dipeptidyl-peptidase-4 (DPP-4) and undergoing renal elimination (19). Therefore, biochemically modified forms of GLP-1, namely GLP-1 receptor agonists (GLP-1RAs), were developed, capable to activate the GLP-1R but having improved bioavailability and extended half-life compared to the native hormone (43). GLP-1RAs were first developed for treating type 2 diabetes and obesity and are now considered an established class of hypoglycemic agents with a very low risk of hypoglycemia (44). Nevertheless, consistent evidence from *in vitro* studies and preclinical models suggests that GLP-1RAs may have broader pharmacological potential. By activating GLP-1R, they lead to cAMP production and consequently to an increased expression and/or activity of receptor tyrosine kinases PI3K/AKT, epidermal growth factor receptor (EGFR), and hypoxia-inducible factor 1-alpha (HIF-1α) (45). Thanks to these mechanisms, GLP-1RAs promote pancreatic β-cell neogenesis, stimulate cell growth and increase insulin synthesis in the β-cells. It was demonstrated that GLP-1RAs reduce ER stress with different mechanisms. In a mouse model with an excess of ER stress in pancreatic β-cells, the mice treatment with the GLP1-RA exendin-4 reduced the expression levels of the ER stress- related molecules immunoglobulin-binding protein (Bip) and C/EBP-homologous protein (CHOP) (46). Similarly, other authors showed that exendin-4 protects β-cells against ER stress by inducing the anti-apoptotic protein JunB and also Bip (47). In addition, they demonstrated that the GLP1-RA inactivated caspase 12 and upregulated Bcl-2 and X-chromosome–linked inhibitor of apoptosis protein, leading to the inhibition of mitochondrial apoptosis. In addition, GLP-1RAs reduce ER stress (46, 47) and lead to favorable metabolic reprogramming and redox homeostasis (19, 48). Several data in the literature demonstrate that GLP-1R activation can also positively affect autophagy (49–53), whose defects have been shown to play a pathogenic role in both type 1 and type 2 diabetes, and in neurodegenerative diseases (54–57). In addition, consistent evidence from *in vitro* studies and preclinical models indicates that GLP-1RAs exert anti-inflammatory effects by modulating the immune system (39). Bendotti et al. reported in detail that GLP-1RAs can modulate the immune system both in mice and humans, independently of the weight loss or glycemic state of the subject. In particular, GLP-1RAs modify the macrophage phenotype toward an anti-inflammatory phenotype and therefore suppress the macrophage secretion of different inflammatory cytokines. Moreover, as a consequence of GLP-1RAs treatment, the inhibition of migration of CD4+ lymphocytes, the decrease in immune cell recruitment, the reduction of monocytes migration and infiltration, and the decrease in the expression of several pro-inflammatory cytokines were determined (39). Hence, thanks to their pleiotropic properties, GLP-1RAs are emerging as suitable for the treatment of diseases associated with chronic inflammation, including type 1 and 2 diabetes, neurodegenerative diseases, atherosclerosis, diabetic nephropathy, asthma, psoriasis, nonalcoholic steatohepatitis (58, 59).

### Uses and potential uses of GLP-1RAs in human diseases

#### Diabetes

GLP-1RAs are recognized as a novel class of anti-diabetic drugs for the treatment of type 2 diabetes and have been suggested as an adjuvant treatment in type 1 diabetes as well (44, 60, 61). In particular, among the GLP-1 RAs, exenatide and liraglutide have been studied in patients with type 1 diabetes (61). GLP-1RAs induce glucose-dependent insulin secretion from pancreatic β-cells, enhance their growth and proliferation, increase their number, inhibit apoptosis, and induce insulin synthesis (62). Another focus of current studies is how non-insulin glucose-lowering medications affect weight loss. Among several anti-diabetic medications, a systemic analysis reveals that GLP1-RAs and dual GLP-1/Gastric inhibitory polypeptide (GIP) agonists such as tirzepatide are most effective at causing weight loss in patients with type 2 diabetes (63).

#### Cardiovascular

GLP-1R are abundantly expressed in the cardiovascular system and by binding them GLP-1RAs may have direct or indirect protective effects by improving cardiac function, increasing the cardiomyocytes activity, decreasing intravascular oxidative stress, inhibiting hepatocyte gluconeogenesis and oxidative stress, and promoting vasodilation (64, 65). Several extensive clinical studies have shown that GLP-1RAs may lower the incidence of cardiovascular events. These studies have been carefully reviewed elsewhere (83, 84), so we won’t go into greater detail about them here.

#### Central nervous system

Recent research has focused on the GLP-1/GLP-1R axis’ protective role against ischemic brain injury. By improving cell survival signaling pathways, lowering ischemia-reperfusion injury, encouraging brain healing, and regulating inflammatory response and oxidative stress, the activation of GLP-1R can decrease the extent of cerebral infarction (66–68). By activating neuronal receptors (69), GLP-1RAs exert beneficial effects on memory function, motor activity, synapse morphology and synaptic function, neurogenesis, apoptosis prevention, and minimizing the chronic inflammation in the brains of various animal models, such as Alzheimer’s Disease, Parkinson’s Disease, amyotrophic lateral sclerosis, multiple sclerosis, or other neurodegenerative diseases (70, 71). However, GLP-1RAs are only studied and not yet used in therapy for neurodegenerative diseases.

#### Asthma

Especially in its advanced phases, asthma is a relatively prevalent chronic lung illness characterized by chronic persistent airway inflammation and airway remodeling that cause incompletely reversible airway blockage. Numerous studies have demonstrated that GLP-1RAs reduce eosinophil production of IL-4, IL-8, and IL-13 and inhibit the PKA/NF-B signaling pathway in animal models of asthma (72). Therefore, for obese patients with asthma, GLP-1RAs therapy may represent a novel add-on therapy (73).

### Preclinical and clinical use of GLP-1RAs in WFS1-SD

The first use of a GLP-1RA in a mouse model of WFS1-SD was reported in 2016 by Sedman et al. (74). In this study, the authors showed that exenatide was capable to lower the blood glucose level and to increase the insulin-to-glucose ratio during the glucose tolerance test, demonstrating the ability of this GLP-1RA to correct the impaired insulin secretion caused by wolframin deficiency. Similar results were obtained by Kondo et al. in 2018, which showed that exenatide could alleviate ER stress by increasing and partially restoring the amount of phosphorylated AMP-activated kinase (p-AMPK), and reducing thioredoxin interacting protein (TXNIP) production in the β-cells of the *Wfs1^−/−^* mice (75). Furthermore, for the first time, these authors described the effect of 24 weeks of treatment with liraglutide in a woman with WFS1-SD, reporting the ability of this GLP-1RA to modulate the β-cell function and to improve glycaemic control in this rare disease. A similar effect was obtained after the administration of the GLP-1RA dulaglutide in a case report of a WFS1-SD patient (76).

In the meanwhile, a research group at the University of Tartu constructed and validated a *Wfs1*-deficient rat. This model of WFS1-SD was described to develop a prominent diabetic phenotype and neurodegeneration of the brainstem and optic nerve very similarly to human patients (77). By using it, it was substantiated that the GLP-1RA liraglutide was effective in preventing the development of glucose intolerance, improving insulin and glucagon secretion control, and reducing ER stress in Langerhans islets in *Wfs1^−/−^* rats (78). Moreover, the treatment resulted capable to delay the onset of diabetes and protecting against the development of optic nerve atrophy and vision loss, even if it did not prevent progressive sensorineural hearing loss (79). The effectiveness of liraglutide in the *Wfs1*-deficient rat model of WFS1-SD was detected not only after early treatment but also when administrated after the onset of several symptoms to mimic a relatively late diagnosis of WFS1-SD in patients (80). In the same rat model, liraglutide has been shown to delay the progression of hyperglycemia and exert neuroprotective effects. In particular, it increased the number of neurons and the neuronal volume in *Wfs1^−/−^* animals’ dorsal nuclei, and decreased ER stress and neuroinflammation in the inferior olive. Finally, liraglutide protected the optic nerve axons from degeneration and counteracted retinal ganglion cell death (80). Recently it was suggested a combination treatment with 7,8-dihydroxyflavone (7,8-DHF) to further improve the liraglutide neuroprotective effect in the rat model of WFS1-SD (81).

Considering the overall results obtained in preclinical models of WFS1-SD and the case report data in a WFS1-SD patient (**Table 1**), IRCCS San Raffaele Hospital in Milan, Italy, started an off-label treatment of liraglutide in pediatric patients with WFS1-SD (18). Recently and for the first time, Frontino et al. reported in detail the follow-up of a small cohort of WFS1-SD patients treated with 1.8mg/day liraglutide for 8-27 months. In this study, four genetically determined WFS1-SD pediatric patients with insulin-dependent diabetes mellitus and optic atrophy were enrolled to obtain preliminary data regarding the safety, tolerability, and efficacy of daily treatment with liraglutide. The authors observed a decrease in insulin requirement, stabilization of neuro-ophthalmological and neurophysiological disease parameters, and no onset of new WFS1-SD-related symptoms at the latest follow-up (18). Accordingly, a very recent case report described the treatment of a WFS1-SD patient with 0.6 mg per day liraglutide in association with the chemical chaperone tauroursodeoxycholic acid (TUDCA) to mitigate ER stress, reduce insulin requirements, and improve glycemic control (82).

**Table 1:** Current effects of the GLP-1RAs use in WFS1-SD.

Species	GLP1-RA	Effects in WFS1-SD	Reference
mouse	exenatide	improving of glycaemic control	Sedman et al. (74)
	exenatide	improving of glycaemic control	Kondo et al. (75)
rat	liraglutide	preventing development of glucose intolerance, improving insulin and glucagon secretion control, reducing ER stress in Langerhans islets	Toots et al. (78)
	liraglutide	delaying onset of diabetes, protecting against optic nerve atrophy and vision loss	Jagomäe et al. (79)
	liraglutide	delaying progression of hyperglycaemia and neuroprotective effects	Seppa et al. (80)
	liraglutide	neuroprotective effect	Seppa et al. (81)
human	dulaglutide	improving of glycaemic control	Scully et al. (76)
	liraglutide	improving of glycaemic control	Kondo et al. (75)
	liraglutide	decreasing of insulin requirement, stabilizating neuro-ophthalmological and neurophysiological disease parameters	Frontino et al. (18)
	liraglutide	improving of the glycaemic control	Png et al. (82)

## Discussion

The numerous beneficial properties of GLP-1RAs and the well-recognized effect in type 2 diabetes encouraged testing GLP-1RAs in animal models of WFS1-SD to investigate if these compounds could be useful in the management of diabetes in this rare disease. All the GLP-1RAs tested for this purpose so far in WFS1-SD, namely exenatide, liraglutide, and dulaglutide (**Table 1**), improved the glycemic control both in rodents and in patients. This effect may depend on the fact that GLP-1 receptor-mediated signaling directly modulates the ER response, leading to the promotion of β-cell adaptation and preventing their apoptosis (46, 83). In addition, it was established an association of genetic variations in the *WFS1* locus with reduced GLP-1-induced insulin secretion and a higher risk of type 2 diabetes (84). Thus, alterations of ER homeostasis deriving from *WFS1* variants could be associated with impaired incretin action and, consequently, β-cell dysfunction (84). Therefore, it is possible to speculate that the activation of the GLP-1 receptor signal using GLP-1RAs could restore the incretin deficiency in WFS1-SD, alleviating insulin insufficiency and aiding glycemic control.

However, in WFS1-SD diabetes is only one of the disease manifestations, being the severe neurological disabilities responsible for the poor prognosis (4). In this regard, evidence highlighting the possible use of GLP-1RAs for the treatment of diseases associated with chronic inflammation and progressive neurodegeneration (38), raised the possibility that GLP-1RAs could counteract also the neurological manifestations in WFS1-SD by impairing neuronal inflammation. This appears a reasonable hypothesis since small peptides GLP-1RAs, including liraglutide and exendin-4, can play a central role and affect the brain because they easily cross the blood-brain barrier upon peripheral administration (19). In this regard, liraglutide protects against optic nerve atrophy and vision loss in the *Wfs1 ^-/-^* rats (79–81) and WFS1-SD patients (18). These liraglutide-mediated neuroprotective effects may derive from its ability in modulating the ER stress response and eliciting ER proteostasis in brain cells (85). Another possible mechanism explaining the beneficial effect of GLP-1RAs in counteracting the progression of neurological manifestations in WFS1-SD is the ability of these molecules to modulate autophagy, whose defects have been shown to play a pathogenic role in neurodegenerative diseases (50, 52).

Recent discoveries highlighted that WFS1-SD is associated with chronic inflammation (11, 86). GLP-1RAs could be useful in WFS1-SD also in this context, due to their ability to modulate the immune system and inflammation by inducing a reduction in the production of proinflammatory cytokines (39).

Overall, current data suggest that GLP-1RAs could effectively be used as therapeutic agents in WFS1-SD. They can improve glycemic control and are promising candidates for delaying neuronal-related symptoms in patients with WFS1-SD. Future perspectives on this regard will be: (i) to verify that liraglutide alters the progression or improves the life expectancy of affected individuals by increasing the number of patients treated with this GLP-1RA; (ii) to reduce the number of administrations and increase the compliance of the patients by investigating if the GLP-1RA with longer half-life semaglutide may have the same effect; (iii) to investigate if the dual GLP-1RA/GIP agonists may provide even more promising results. In fact, other incretins, such as GIP, have also shown neuroprotective effects in animal models of neurodegenerative diseases. Newer dual GLP-1/GIP receptor agonists or so-called twincretins have also shown protective effects in murine models of AD and PD (87). These results are encouraging and suggest that the development of such dual agonists for the treatment of other neurodegenerative diseases such as WFS1-SD may be very promising.

## Author contributions

All authors listed have made a substantial, direct, and intellectual contribution to the work and approved it for publication.
